# Hypolipidemic potential of squid homogenate irrespective of a relatively high content of cholesterol

**DOI:** 10.1186/1476-511X-13-165

**Published:** 2014-10-29

**Authors:** Yasuo Nagata, Youhei Noguchi, Shizuka Tamaru, Koichi Kuwahara, Akira Okamoto, Kazuhito Suruga, Kazunori Koba, Kazunari Tanaka

**Affiliations:** Department of Nutrition, University of Nagasaki, 1-1-1 Manabino, Nagayo-cho, Nishisonogi-gun, Nagasaki 851-2195 Japan; Food Science and Technology Section, Nagasaki Prefectural Institute of Fisheries, 1551-4 Taira-machi, Nagasaki, 851-2213 Japan; Center for Industry, University and Government Cooperation, Nagasaki University, 1-14 Bunkyo-machi, Nagasaki, 852-8521 Japan

**Keywords:** Squid homogenate, Hypocholesterolemic, Hypotriglyceridemic, Bile acids, Shellfish

## Abstract

**Background:**

Our previous study has shown that regardless of a relatively high amount of cholesterol, squid homogenate lowers serum and hepatic cholesterol in animals. Since this work, we have developed a new method to inhibit autolysis of squid proteins with sodium citrate. This study aims to investigate how squid homogenate prepared with sodium citrate affects lipid metabolism in Sprague–Dawley rats at the molecular level.

**Methods:**

We prepared squid homogenate with sodium citrate to inhibit autolysis of squid protein. In Experiment 1 (Exp. 1), rats were given a cholesterol-free control diet or a squid diet, with squid homogenate added at the level of 5% as dietary protein for 4 weeks. Blood, the liver and adipose tissue were taken after 6 hours fasting. Serum and hepatic lipids and activities of enzymes related to lipid metabolism were measured. In Experiment 2 (Exp. 2), the above-mentioned diets had cholesterol added at the level of 0.1% and given to rats. Lipid parameters, enzyme activities, and gene expression of proteins involved in lipid metabolism in the liver and the small intestine were determined. In addition, feces were collected for two days at the end of Exp. 2 to measure fecal excretion of steroids.

**Results:**

In Exp.1, serum triglyceride and cholesterol were ~50% and ~20% lower, respectively, in the squid diet-fed rats than in the control diet-fed animals while hepatic cholesterol was ~290% higher in the squid diet-fed rats. When cholesterol was included into the diets (Exp. 2), serum lipids were significantly lower in the squid group while no difference of hepatic lipid was seen between two groups. Activities of hepatic lipogenic enzymes were significantly lower in rats on the squid diet while the enzyme responsible for fatty acid oxidation was not modified (Expt. 1 and 2). Hepatic level of mRNA of microsomal triglyceride transfer protein was significantly lower in the squid group. In the small intestine, the squid diet exhibited significantly lower gene expression of proteins involved in fatty acid transport and cholesterol absorption. Fecal secretion of acidic steroids, but not neutral steroids, was higher in rats fed the squid diet than in those fed the control diet.

**Conclusion:**

These results imply that newly-developed squid homogenate has hypolipidemic potential primarily through decreased absorption of bile acids in the small intestine and suppressed lipogenesis in the liver.

## Background

Besides genetic factors, dietary factors have been known to be associated with increased cholesterol in blood, consequently modifying risk of coronary heart diseases (CHD) [1]. Among them, the link between fish consumption and CHD has been extensively studied [2–5]. There is little doubt that fish oil, especially omega-3 fatty acids, is effective in improving lipid profile and subsequently reducing risk of CHD [6]. Studies have suggested that fish oil displays a wide spectrum of biological activities, including hypotriglyceridemic, antiinflammatory and antithrombotic activities [7]. However, the role of other constituents in fish such as protein in development of various diseases has been less conclusive compared to those of fish oil, although fish proteins appear to be less hyperlipidemic than animal proteins [8–11]. Some shellfish such as crab, squid and shrimp have a relatively high content of cholesterol [12], which often discourages subjects with hypercholesterolemia from eating them. However, a few studies have been carried out to see how shellfish affect lipid metabolism [12, 13]; some shellfish favorably affect cholesterol metabolism, increasing HDL_2_ cholesterol or HDL_2_-/HDL_3_-cholesterol ratio. Squid contains a relatively low amount of fat (4.3%) and a high amount of cholesterol (~100 mg/100 g dry matter) [14]. Nevertheless, we previously reported that in mice and rats, squid reduced serum and liver lipids in the presence and absence of dietary cholesterol [14, 15]. The fact that defatted squid also suppressed serum lipids in rats is indicative of hypolipidemic action of squid proteins since defatted squid contains >85% protein as dry matter [15]. When caught in the sea, squid proteins are easily degraded because of autolysis. Thus, elastic thermal gel of squid could not be produced, and commercial value of squid could be lost for fish cake processing. We have tried to diminish autolysis and found that sodium citrate inhibits autolysis of squid mantle muscle [16]. Therefore, squid homogenate prepared with sodium citrate could contain more intact proteins compared to squid homogenate used in the previous study [15]. The aim of this study was to see if newly-developed squid homogenate lowers lipid levels in blood and the liver, and to determine how squid homogenate modifies lipid metabolism at the molecular level.

We here show that squid homogenate reduces serum lipids and hepatic lipogenic enzyme activity, alters gene expression of lipid-related proteins, and modifies lipid metabolism at the molecular level in rats.

## Results

### Experiment 1

As shown in Table 1, body weight gain and liver weight were not affected by the diets while the squid diet animals had significantly lower food intake and perirenal adipose tissue weight compared to the control diet animals. Serum levels of triglyceride, cholesterol and phospholipid were significantly lower in rats fed the squid diet compared to those fed the control diet (Table 2); triglyceride level was ~50% lower in rats on the squid diet. On the other hand, hepatic cholesterol level was significantly higher in rats on the squid diet when compared to those on the control diet. Activities of enzymes involved in lipid metabolism are described in Table 3. Feeding the squid diet showed a trend toward lower activity of lipogenic enzymes with significantly lower malic enzyme (ME), glucose-6-phosphate dehydrogenase (G6PDH) and phosphatidate phosphohydrolase (PAP). In contrast, carnitine palmitoyltransferase (CPT), the rate-limiting enzyme of fatty acid oxidation, in the liver and brown adipose tissue was not altered by the diets.

**Table 1:** **Effects of the diets on body weight, food intake and tissue weight (Exp.1)**

	Control			Squid		
Body weight						
Final (g)	396	±	14	401	±	8
Gain (g/day)	4.40	±	0.31	4.80	±	0.10
Food intake (g/day)	23.5	±	0.6	20.4	±	0.6*
Tissue weight						
Liver weight (g/100 g BW)	4.19	±	0.15	3.93	±	0.08
White adipose tissue weight (g/100 g BW)						
Perirenal	1.88	±	0.20	0.89	±	0.06*
Epididymal	1.14	±	0.14	1.59	±	0.16
Mesenteric	0.98	±	0.06	0.89	±	0.06
Total	3.99	±	0.30	3.37	±	0.27
Brown adipose tissue weight (g/100 g BW)	0.142	±	0.013	0.121	±	0.009

Data are mean ± SE (n = 6).

*Significantly different from the control group at *p* < 0.05.

**Table 2:** **Effects of the diets on the concentrations of serum hepatic lipids (Exp.1)**

	Control			Squid		
Serum						
Triglyceride (mg/dL)	205	±	12	106	±	10*
Phospholipid (mg/dL)	171	±	10	100	±	1*
Cholesrerol (mg/dL)	102	±	5	81.3	±	1.6*
Liver						
Triglyceride (mg/g)	29.3	±	3.2	45.3	±	5.3
Phospholipid (mg/g)	14.7	±	0.5	14.4	±	0.2
Cholesrerol (mg/g)	3.61	±	0.24	10.5	±	1.2*

Data are mean ± SE (n = 6).

*Significantly different from the control group at *p* < 0.05.

**Table 3:** **Effects of the diets on activities of hepatic enzymes involved in lipid metabolism (Exp.1)**

	Control			Squid		
Lipogenic enzyme (nmol/min/mg protein)						
Fatty acid synthase	7.69	±	1.73	4.12	±	1.58
Malic enzyme	16.5	±	2.9	8.9	±	1.7*
Glucose-6-phosphate dehydrogenase	25.2	±	3.6	11.6	±	2.7*
Phosphatidate phosphohydrolase	43.7	±	9.04	22.3	±	2.85*
Fatty acid-oxidizing enzyme (nmol/min/mg protein)						
Carnintine palmitoyltransferase						
In the liver	2.88	±	0.33	2.25	±	0.28
In brown adipose tissue	7.52	±	0.85	8.01	±	0.79

*Significantly different from the control group at *p* < 0.05.

### Experiment 2

**Figure 1:**
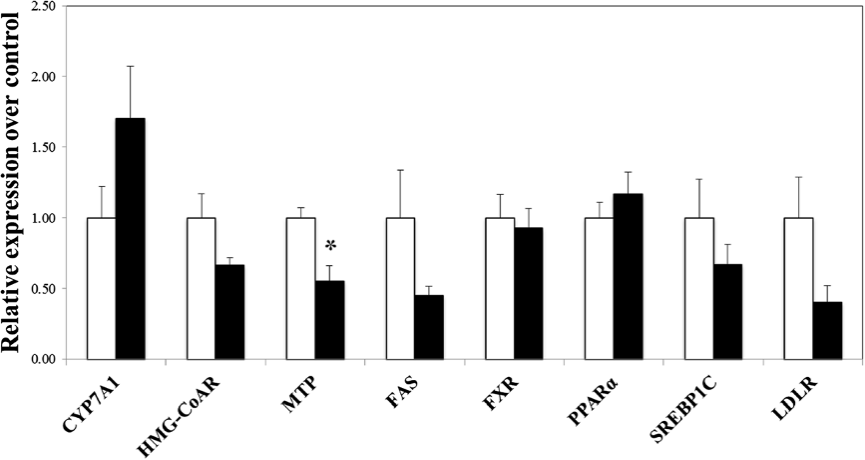
**Effects of the diets on hepatic mRNA expression of lipid metabolism-related genes.** Data are mean ± SE (n = 6-7). □: the control diet, ■: the squid diet. *: Significantly different from the control group at *p* <0.05.

**Figure 2:**
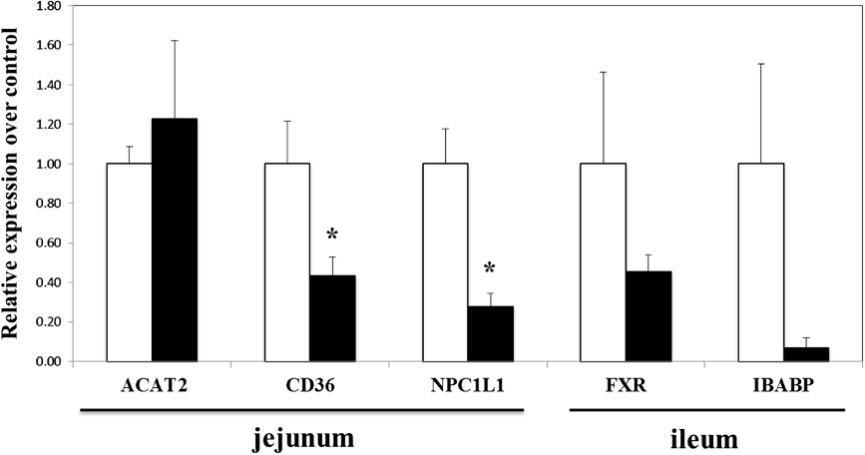
**Effects of the diets on intestinal mRNA expression of lipid metabolism-related genes.** Data are mean ± SE (n = 6-7). □: the control diet, ■: the squid diet. *: Significantly different from the control group at *p* <0.05.

To make the level of dietary cholesterol in the control diet equivalent to that in the squid diet, cholesterol was added to diets at the level of 0.1%. As shown in Table 4, no effect of the diets was seen on body weight gain, food intake, liver weight and adipose tissue weight. Serum levels of triglyceride, phospholipid and cholesterol were ~45%, ~35% and ~17% lower, respectively, in the squid group compared to the control group (Table 5). However, there was no significant difference in hepatic concentrations of triglyceride, cholesterol and phospholipid between two diets. The squid diet markedly affected hepatic lipogenic enzyme activities (Table 6); animals displayed significantly lower enzyme activities of fatty acid synthase (FAS) and PAP compared to the control diet. However, the diets did not change CPT activity. As shown in Table 7, fecal weight and fecal excretion of fatty acid were not influenced by the diets whereas fecal excretion of acidic steroids was significantly higher in the squid group than the control group with no change in neutral steroids. Figure 1 illustrates expression of genes involved in hepatic lipid metabolism. The expression of 3-hydroxy-3-methylglutaryl-coenzyme A reductase (HMG-CoAR), essential for cholesterol synthesis, was non-significantly down-regulated by the squid diet (*p* =0.1) while that of CYP7A1, the rate-limiting enzyme in bile acid synthesis, was up-regulated (*p* =0.08). Although fecal excretion of bile acid, a ligand for farnesoid X receptor (FXR), was significantly enhanced by the squid diet, mRNA of hepatic FXR was not changed by the squid diet. Gene expression of enzyme responsible for fatty acid synthesis, FAS, was lower in the squid group than in the control group without a significant difference (*p* =0.17). The squid diet showed a significantly lower hepatic expression of microsomal triglyceride transfer protein (MTP), essential for very low density lipoprotein (VLDL) assembly/secretion (*p* <0.05). As shown in Figure 2, gene expression of jejunum Niemann- Pick C1-like 1 (NPC1L1), a critical mediator of intestinal dietary cholesterol absorption, was significantly lower in rats on the squid diet compared to those on the control diet (*p* <0.05), but the diets did not significantly alter acyl-coenzyme A:cholesterol acyltransferase 2 (ACAT2), the rate-limiting enzyme in intestinal cholesterol trafficking. In addition, mRNA of fatty acid translocase (CD 36), fatty acid transporter, in the jejunum was significantly lower in rats fed the squid diet compared to those fed the control diet (*p* <0.05). Gene expression of ileal bile acid binding protein (IBABP) (*p* =0.09), bile acid transporter, was lower in the squid group without a significant difference (Figure 2).

**Table 4:** **Effects of the diets on body weight, food intake and tissue weight (Exp.2)**

	Control			Squid		
Body weight						
Final (g)	351	±	17	352	±	8
Gain (g/day)	7.49	±	0.40	7.52	±	0.27
Food intake (g/day)	20.5	±	0.8	21.1	±	0.9
Tissue weight						
Liver weight (g/100 g BW)	4.29	±	0.08	4.00	±	0.14
White adipose tissue weight (g/100 g BW)						
Perirenal	1.45	±	0.16	1.29	±	0.27
Epididymal	1.08	±	0.06	1.01	±	0.15
Mesenteric	1.06	±	0.08	0.923	±	0.161
Total	3.58	±	0.28	3.22	±	0.56
Brown adipose tissue weight (g/100 g BW)	0.153	±	0.011	0.156	±	0.009

Data are mean ± SE (n = 6-7).

**Table 5:** **Effects of the diets on the concentrations of serum and hepatic lipids (Exp.2)**

	Control			Squid		
Serum						
Triglyceride (mg/dL)	177	±	24	97.7	±	8.7*
Phospholipid (mg/dL)	153	±	9	100	±	8*
Cholesrerol (mg/dL)	78.3	±	2.9	65.3	±	1.3*
Liver						
Triglyceride (mg/g)	75.4	±	4.1	70.4	±	9.8
Phospholipid (mg/g)	17.0	±	0.5	17.3	±	0.2
Cholesrerol (mg/g)	4.99	±	0.39	4.15	±	0.35

Data are mean ± SE (n = 6-7).

*Significantly different from the control group at *p* < 0.05.

**Table 6:** **Effects of the diets on activities of hepatic enzymes involved in lipid metabolism (Exp.2)**

	Control			Squid		
Lipogenic enzyme (nmol/min/mg protein)						
Fatty acid synthase	4.67	±	0.99	2.40	±	0.58*
Malic enzyme	15.7	±	2.7	9.70	±	1.03
Glucose-6-phosphate dehydrogenase	8.51	±	1.43	4.12	±	0.50
Phosphatidate phosphohydrolase	5.91	±	0.32	4.82	±	0.20*
Fatty acid-oxidizing enzyme (nmol/min/mg protein)						
Carnintine palmitoyltransferase						
In the liver	2.12	±	0.42	2.36	±	0.58
In brown adipose tissue	5.95	±	0.86	4.55	±	1.16

Data are mean ± SE (n = 6-7).

*Significantly different from the control group at *p* < 0.05.

**Table 7:** **Effects of the diets on fecal fatty acid and steroid excretion (Exp.2)**

	Control			Squid		
Dry feces weight (g/2 day)	4.27	±	0.33	4.41	±	0.26
Fatty acid (mg/2 day)	69.4	±	8.3	61.2	±	3.3
Neutral steroids (mg/2 day)	8.27	±	0.83	8.50	±	0.76
Acidic steroids (mg/2 day)	24.4	±	2.3	33.7	±	4.3*
Total steroids (mg/2 day)	31.8	±	2.1	42.2	±	4.3*

Data are mean ± SE (n = 6-7).

*Significantly different from the control group at *p* < 0.05.

## Discussion

It has long been known that dietary protein affects cholesterol level in blood; animal proteins such as casein are hypercholesterolemic while vegetable proteins such as soybean are hypocholesterolemic [8]. Studies have previously suggested that hypocholesterolemic action of soy protein is primarily due to peptides [17, 18]. It’s been reported that peptides from soybean reduce solubility of cholesterol in micelle [18]. Some peptides appeared to bind to bile acids in the small intestine, thereby decreasing bile acids returning to the liver [19, 20]. Cholesterol is then converted to bile acids to compensate for the decreased bile acid pool, lowering cholesterol level in serum and/or the liver. Many studies have since tried to pinpoint peptides responsible for cholesterol-lowering action [21–23]. Studies investigating dietary proteins and peptides with hypocholesterolemic action have used mainly vegetable proteins such as rice and soybean [20, 22, 24]. On the other hand, a few studies have been explored using shellfish [12–15]. Squid homogenate prepared with sodium citrate in the current study retained heavy chain myosin more intact as compared to that prepared by simply mincing squid [25]. Seventy five percent of dietary protein (casein) was replaced with squid homogenate in the previous study [15] while a quarter of it was substituted with sodium citrate-treated squid homogenate in the current study. Therefore, although we did not directly compare effects of sodium citrate-treated squid homogenate on lipid parameters with those of squid homogenate previously prepared by Tanaka et al. [15], the present study suggests that squid homogenate prepared by the new method may have more hypolipidemic potential than that previously prepared. This similar hypolipidemic effect despite using a smaller amount of squid homogenate may be that intact proteins simply bind to bile acids more than autolyzed proteins. Alternatively, peptides responsible for bile acid binding may be partly decomposed during homogenate preparation in the previous study. Tanaka et al. [15] have shown that defatted squid lowers serum and hepatic cholesterol and hepatic triglyceride accompanied with enhanced bile acid secretion into feces in rats, indicating that protein, but not lipids, is responsible for lipid-lowering action of squid. However, in a mouse study, defatted squid-fed mice had higher levels of serum lipids compared to the control diet-fed animals [14]. The discrepancy is probably due to species difference and different dietary composition; the mouse study used lard as fat source and sucrose as carbohydrate source while soybean oil and a mixture of cornstarch and sucrose were added in our rat study. Studies using various types of fish protein did not clearly show changes in serum triglyceride in rats although lowered cholesterol was seen in the liver [10, 11]. Rice and pea proteins had hypotriglyceridemic action in rats as well as hypocholesterolemic action [26, 27] while another study with vegetable proteins showed hypocholesterolemic action, but not hypotriglyceridemic action [28]. It thus seems likely that dietary proteins derived from different sources modify lipid metabolism differently.

Then, to clarify mechanisms responsible for triglyceride-lowering action of squid homogenate in more detail, we measured activities of hepatic enzymes involved in lipid metabolism. Whether or not cholesterol was in the control diet, animals fed the squid diet exhibited a lower activity of enzymes synthesizing fatty acids including FAS, ME and G6PDH (Tables 3 and 6). This corroborates results from the study with defatted squid in which activities of FAS, ME and G6PDH were reduced [15]. On the other hand, CPT, the rate-limiting enzyme in fatty acid oxidation, was not changed by the diets, indicating reduced serum triglyceride to be at least in part due to suppression of fatty acid synthesis, but not enhancement of fatty acid oxidation. Furthermore, gene expression of MTP, responsible for VLDL assembly/secretion, was significantly lower in the squid group, suggesting that squid homogenate may alter hepatic secretion of VLDL triglyceride. Collectively, these results imply that squid homogenate modulates synthesis and secretion of triglyceride in the liver at the molecular level. Since activities of lipoprotein lipase (LPL) and hepatic lipase (HL) were enhanced by rice protein [26], dietary protein may stimulate clearance of triglyceride from the circulation. It is therefore of interest to see how squid homogenate affects secretion of triglyceride from the liver, clearance of triglyceride from the circulation, and uptake of triglyceride in peripheral tissues such as muscle in further studies. Hepatic triglyceride levels in Exp. 2 were higher than those in Exp. 1 in the present study. Although adding cholesterol diets might have increased hepatic triglyceride as observed by Tanaka et al. [15], the precise mechanism is currently unclear. However, since there was no difference of hepatic triglyceride between the control group and the squid group, and since weight of the liver and adipose tissue tended to be lower in squid-fed rats, the levels of hepatic triglyceride in the squid group may not be detrimental.

Serum cholesterol in rats fed the squid diet was significantly lower than in those fed the control diet (Tables 2 and 5). In Exp. 1, higher hepatic cholesterol in the squid group was seen compared to that in the control group (Table 2). This increased level of cholesterol in the liver is not derived from hepatic cholesterol synthesis but, rather, probably is due to the small amount of endogenous cholesterol in squid homogenate because no difference of hepatic cholesterol was found in Exp.2 in which cholesterol levels in the diets were the same. In Table 7, fecal excretion of neutral steroids (including cholesterol) was not modified by the diets, suggesting that decreased cholesterol in blood may not be due to diminished absorption of dietary cholesterol in the small intestine. On the other hand, feeding the squid diet gave a significantly lower gene expression of intestinal CD36 and NPC1L1, both of which are necessary for intestinal cholesterol trafficking (Figure 2). By contrast, fecal excretion of acidic steroids (bile acids) was significantly higher in the squid group accompanied with a non-significant decrease in mRNA of IBABP (*p* =0.1) (Table 7 and Figure 2). Thus, hypocholesterolemic action of squid is likely to be secondary to enhanced fecal excretion of bile acid rather than an inhibition of cholesterol absorption in the small intestine. Our results are not in agreement with those by Childs et al. showing that some shellfish exerted lower cholesterol absorption in humans [13]. Different source of shellfish proteins and species difference may explain the contradiction. Gene expression of CYP7A1 tended to be higher without a significant difference (*p* =0.08, Figure 1). Decreased absorption of bile acids might have induced hepatic mRNA for CYP7A1, probably stimulating conversion of cholesterol to bile acid. Though gene expression of CYP7A1 is known to be regulated by FXR [29], hepatic FXR gene expression was not modulated by squid homogenate (Figure 1). It has been reported that bile acids in the small intestine activate intestinal FXR, and in turn FXR stimulates FGF15/19 production in the small intestine, which signals to the liver to repress CYP7A1 expression [30]. Since bile acids were excreted into feces more by the squid diet, it is likely that decreased absorption of bile acids in intestinal cells might have repressed ileum FXR gene expression (Figure 2), consequently enhancing bile acid synthesis in the liver through decreased FGF15. Thus, although FGF15 was not measured in the present study, FXR/FGF15 intestine-liver pathway may be at least in part responsible for hepatic increased level of CYP7A1. Further studies are needed to prove our hypothesis as, most likely due to a large standard error, no clear difference of gene expression of CYP7A1 in the liver and FXR and IBABP in the small intestine was observed. Treatment with cholestyramine, bile acid sequestrant, has been reported to lower serum FGF19, but simultaneously elevate blood bile acid levels in humans probably via increased synthesis of bile acid [31]. However, bile acid sequestrant concomitantly increased plasma triglyceride in some studies [32, 33]. Similar to cholestyramine, squid homogenate stimulated fecal excretion of bile acid, but indeed decreased serum triglyceride level in our study (Tables 2 and 5). Reasons for the discrepancy on serum triglyceride between squid homogenate and cholestyramine remain unclear at present. Since Kim et al. showed that the small intestine plays a much more prominent role in repressing CYP7A1 expression than the liver [34], further detailed studies on an intestinal role in bile acid and lipid metabolism in squid-fed rats are warranted.

There is the discrepancy of effects of squid on hepatic lipid levels between our study and the previous study [15]. Tanaka et al. used 0.5% cholesterol- and defatted squid-supplemented diets and higher level of squid protein (~15%), and showed that in addition to decreased lipids in blood, hepatic levels of cholesterol and triglyceride were also lower by feeding squid [15]. This discrepancy on hepatic lipid might have been largely attributed to the difference of dietary composition, but effects of defatting, which may remove factors affecting lipid metabolism, cannot be ruled out.

It has been known that some peptides derived from soybean are capable of binding to bile acids in the small intestine [18, 19, 21]; vegetable proteins and their peptides bind to cholesterol and/or bile acids in the small intestine and stimulate fecal excretion of steroids, resulting in lowered blood and liver cholesterol. Although hypolipidemic effects of vegetable proteins and peptides were not constant [19–21], some peptides from vegetable proteins appears to increase mRNA of CYP7A1, LPL and low density lipoprotein receptor (LDLR), indicating the possibility that peptides may lower cholesterol by enhanced bile acid synthesis through CYP7A1, and decrease triglyceride by enhanced clearance through LPL and LDLR [26–28]. In addition, since, as mentioned above, a lower amount of homogenate was required to induce hypolipidemic action in the current study than in the previous study [15], it is worth finding peptides responsible for hypolipidemic action from squid to develop novel functional foods. Studies are currently under way in our laboratory to pinpoint peptides.

## Conclusion

The present study shows that regardless of the presence or absence of dietary cholesterol in the diets, squid homogenate is capable of lowering serum lipids in rats. Newly-developed squid homogenate favorably alters lipid metabolism by changes in hepatic fatty acid synthesis in addition to lipid handling in the small intestine. Decreased secretion of lipids as VLDL from the liver may also be involved in serum lipid levels as suggested by down-regulated gene expression of MTP. The squid diet supplemented with or without cholesterol significantly lowered serum cholesterol, but serum triglyceride was affected to a greater extent. These results could provide new dietary strategies to maintain healthy levels of serum cholesterol and triglyceride. Further studies are definitely merited to figure out the detailed mechanism responsible for hypolipidemic action of squid and responsible peptide fraction.

## Methods

### Animal experiments

Four week-old male Sprague–Dawley rats were purchased from Japan SLC (Shizuoka, Japan), housed individually in stainless-steel cages under a controlled atmosphere (temperature, 22 ± 1°C, humidity, 55 ± 5%, light cycle, 0800–20:00), fed a commercial pellet diet (CE-2, Clea Japan, Tokyo) for a week, and then given the experimental diets. Animal studies were carried out under the Guidelines for Animal Experiments of University of Nagasaki (Nagasaki, Japan), and Law no.105 and Notification no. 6 of the Government of Japan.

### Preparation of homogenated squid muscle with sodium citrate

Approximately 310 g in body weight of Japanese common squid (*Todarodes pacificus*) with 25 cm in mantle length was caught off the coast of Nagasaki prefecture, Japan. The squid were gutted and skinned immediately. Mantle muscle was minced and extruded with a diameter of 1.2 mm. The minced mantle muscle was treated with sodium citrate at the concentration of 2% (w/w) and blended as described elsewhere [16]. Then, the minced squid muscle was lyophilized and powdered before use. The electrophoretic pattern of sodium citrate-treated squid proteins was well characterized in comparison with non-treated squid homogenate [25]. The level of protein, carbohydrate, fat, mineral, cholesterol, and water in squid homogenate was 61.4%, 0.1%, 2.2%, 18.3%, 0.65% and 17.4%, respectively, as dry matter.

### Experimental diet

#### Experiment 1

To see if squid homogenate has potential in reducing blood lipids in comparison with a casein-based AIN-93G cholesterol-free diet, rats were assigned to 2 groups of 6 animals. The dietary composition is described in Table 8. The proportion of energy derived from protein, fat and carbohydrate was 20%, 16% and 64%, respectively, in the control diet, and 21%, 17% and 62%, respectively, in the squid diet. Animals were fed the diets for 4 weeks and sacrificed after 6 hours fasting. Blood was withdrawn and was centrifuged at 1500 g for 20 min after clotting. The liver and adipose tissue were excised, frozen immediately in liquid nitrogen and stored at −80°C before analysis. Serum and hepatic lipid concentrations were measured as described below. Activity of hepatic enzymes involved in lipid metabolism was also determined.

**Table 8:** Nutrient composition in the diets *(g)

	Experiment 1		Experiment 2	
	Control	Squid	Control	Squid
Casein*	20.0	15.0	20.0	15.0
Squid homogenate**	-	8.143	-	8.143
α-Com starch	13.2	13.2	13.2	13.2
Soybean oil	7.0	7.0	7.0	7.0
Sucrose	10.0	10.0	10.0	10.0
Cellulose	5.0	5.0	5.0	5.0
Mineral mix AIN-93G	3.5	3.5	3.5	3.5
Vitamin mix AIN-93G	1.0	1.0	1.0	1.0
Tert-Butylhydroquinone	0.0014	0.0014	0.0014	0.0014
Choline bitartrate	0.25	0.25	0.25	0.25
L-Cystine	0.30	0.30	0.30	0.30
Cholesterol	-	0.0529***	0.10	0.10****
β-Com starch	39.75	36.61	39.65	36.56
Total (g)	100.0	100.0	100.0	100.0

*Purchased from Wako Pure Chemical Industries. (Osaka, Japan).

**Squid homogenate was equivalent to 5 g of protein.

***Endogenous cholesterol in squid.

****Endogenous plus dietary cholesterol.

#### Experiment 2

To see if squid homogenate has potential in reducing blood lipid in the presence of dietary cholesterol, rats were given one of two diets prepared as described in Exp. 1 except that cholesterol was added at the level of 0.1% and 0.047% to the control diet and the squid diet, respectively, to provide the same amount of dietary cholesterol. Animals fed the diets for 4 weeks were sacrificed after 6 hours fasting. In addition to the liver and adipose tissue, the entire small intestine was collected, flushed with ice-cold saline, divided into two segments designated as jejunum and ileum, respectively, and stored in −80°C before use. Serum and hepatic lipid concentrations and activity of enzymes involved in lipid metabolism were also determined as in Exp. 1. Gene expression of proteins responsible for lipid metabolism in the liver and the small intestine was determined by quantitative real-time PCR, as described below. Feces were collected for two days before sacrifice, and fecal excretion of fatty acid and steroids was measured.

### Serum and tissue biochemical analyses

Serum lipids were assayed enzymatically using commercial kits (Cholesterol E-test, Triglyceride E-test, Phospholipid C-Test, Wako Pure Chemical Industries, Osaka, Japan). Liver lipids were extracted by the method of Folch et al. [35], and then the concentrations of cholesterol, phospholipid, and triglyceride were measured enzymatically.

### Analyses of enzyme activity and gene expression of lipid metabolism-related proteins

The liver was excised, frozen immediately in liquid nitrogen and stored at −80°C before use. A small aliquot of the excised liver was homogenized in 6 volumes of 0.25 M sucrose solution containing 10 mM Tris–HCl and 1 mM EDTA (pH7.4). After sedimentation of the nuclei fraction, the supernatant was centrifuged to sediment mitochondria at 12,000 g for 10 min. Then, the supernatant was again centrifuged at 100,000 g for 60 min to precipitate microsomes, and the remaining supernatant was used as cytosol fraction. The mitochondrial and microsomal pellets were resuspended in the same 0.25 M sucrose solution. Activities of cytosol fatty acid synthase (FAS) [36] and glucose-6-phosphate dehydrogenase (G6PDH) [37] and malic enzyme (ME) [38] were measured. Microsomal phosphatidate phosphohydrolase (PAP) [39] and mitochondrial carnitine palmitoyltransferase (CPT) [40] were also determined. Interscapular brown adipose tissue was collected and homogenized as described above, and mitochondrial CPT activity was measured. Protein was assayed by the method of Lowry et al. [41].

Total RNA was extracted from the small intestine and the liver using RNA extraction reagent (RNAiso Plus, Takara Bio, Shiga, Japan) according to the manufacturer’s instructions. Total RNA (1 μg) was reverse-transcribed to synthesize cDNA with reverse-transcription kit (PrimeScript ®RT Master Mix, Takara Bio, Shiga, Japan). Real time PCR was performed on a 7300 Real-Time PCR system (Applied Biosystems, CA, USA) using the manufacturer’s standard protocol. The PCR reaction mixture was prepared using THUNDERBIRD SYBR qPCR Mix (TOYOBO, Osaka, Japan). The messenger RNA (mRNA) levels, measured relative to 36B4 mRNA levels used as an internal control, were determined using the 2 − ΔΔCt method. The primers used in this study are listed in Table 9.

**Table 9:** **Sequences of the primers used for real-time PCR**

	Forward	Reverse
ACAT2	5′TCATGCTGTCCTCATCTTCTTC-3′	5′CACCAGTCCCGGTAGAACATTC-3′
CD36	5′CGAAGGCTTGAATCCTAACGAA-3′	5′-GTTGACCTGCAGTCGTTTTGC-3′
CYP7A1	5′TACTTCTGCGAAGGCATTTGG-3′	5′-ATCTCCCTGGAGGGTTTTGG-3′
FAS	5′CGCTCGGCATGGCTATCT-3′	5′CTCGTTGAAGAACGCATCCA-3′
FXR	5′TGCAGAGAAAGGTGCGAAGTT-3′	5′CGTCTCCATGAGGGTTTCCA-3′
HMG-CoAR	5′TGCAGAGAAAGGTGCGAAGTT-3′	5′CGTCTCCATGAGGGTTTCCA-3′
IBABP	5′CGCCTGGGTGTGCGAAGTT-3′	5′AAGACTGGGACCAGGTGAAGTTC-3′
LDLR	5′GATCCCCAACCTGAGAATGTG-3′	5′CACGGCGCTGTAGATCTTTCTC-3′
MTP	5′-CCTAGACATTCTACTCGGGTTCTG-3′	5′AACCACCTGGCTACCGTGAA-3′
NPC1L1	5′ATCTTAACTGTCGGATCCACAAAAA-3′	5′-AACCTGATGGCATTGTGAGACAT-3′
PPARα	5′CGGATACTTCTTCAACTGTTAAA-3′	5′-CGGATACTTCCTTCAACTGCTTAAA-3′
SREBP-1c	5′GACCGACATCGAAGGTGAAGTC-3′	5′-TTGGTTGTTGATAAGCTGAAGCA-3′

### Fecal lipid analyses

In Exp 2, feces were collected for 2 days before sacrifice, lyophilized, and ground. A small aliquot of feces was extracted by the method of Ikeda et al. [42] and measured for lipid excretion with gas chromatography. Neutral and acidic steroids were measured by 5α-cholestane and 23-nordeoxycholic acid as internal standards, respectively. An excretion of fatty acid into feces was determined by a titration method [43].

### Statistical analysis

Statistical analysis was done by Independent *t-*test using IBM SPSS Statistics 20.0. *P* <0.05 was considered significant.

## Abbreviations

ACAT2: Acyl-coenzyme A:cholesterol acyltransferase 2
BW: Body weight
CHD: Coronary heart diseases
CD36: Fatty acid translocase
CPT: Carnitine palmitoyltransferase
CYP7A1: Cytochrome P450 7A1
FAS: Fatty acid synthase
FGF: Fibroblast growth factor
FXR: Farnesoid X receptor
G6PDH: Glucose-6-phosphate dehydrogenase
HL: Hepatic lipase
HMG-CoAR: 3-hydroxyl-3-methylglutaryl-coenzyme A reductase
IBABP: Ileal bile acid binding protein
LDL: Low density lipoprotein
LDLR: Low density lipoprotein receptor
LPL: Lipoprotein lipase
MTT: Microsomal triglyceride transfer protein
NPC1L1: Niemann-Pick C1- Like 1
PAP: Phosphatidate phosphohydrolase
PPARα: Peroxisome proliferator-activated receptor α
SREBP-1c: Sterol regulatory element binding protein-1c
VLDL: Very low density lipoprotein.
